# A model to assess the efficacy of vaccines for control of liver fluke infection

**DOI:** 10.1038/srep23345

**Published:** 2016-03-24

**Authors:** Joanne Turner, Alison Howell, Cathy McCann, Cyril Caminade, Roger G. Bowers, Diana Williams, Matthew Baylis

**Affiliations:** 1 Department of Epidemiology and Population Health, Institute of Infection and Global Health, University of Liverpool, Leahurst Campus, Chester High Road, Neston, CH64 7TE, UK; 2 Department of Infection Biology, Institute of Infection and Global Health, Liverpool Science Park IC2, 146 Brownlow Hill, Liverpool, L3 5RF, UK; 3 Department of Epidemiology and Population Health, Institute of Infection and Global Health, University of Liverpool, Waterhouse Building, Liverpool, L69 3GL, UK; 4 Department of Mathematical Sciences, University of Liverpool, Mathematical Sciences Building, Liverpool, L69 7ZL, UK; 5 NIHR Health Protection Research Unit in Emerging and Zoonotic Infections, Liverpool, L69 7BE, UK

## Abstract

*Fasciola hepatica*, common liver fluke, infects cattle and sheep causing disease and production losses costing approximately $3billion annually. Current control relies on drugs designed to kill the parasite. However, resistance is evident worldwide and widespread in some areas. Work towards a vaccine has identified several antigens of *F. hepatica* that show partial efficacy in terms of reducing worm burden and egg output. A critical question is what level of efficacy is required for such a vaccine to be useful? We have created the first mathematical model to assess the effectiveness of liver fluke vaccines under simulated field conditions. The model describes development of fluke within a group of animals and includes heterogeneity in host susceptibility, seasonal exposure to metacercariae and seasonal changes in temperature affecting metacercarial survival. Our analysis suggests that the potential vaccine candidates could reduce total fluke burden and egg output by up to 43% and 99%, respectively, on average under field conditions. It also suggests that for a vaccine to be effective, it must protect at least 90% of animals for the whole season. In conclusion, novel, partial, vaccines could contribute substantially towards fasciolosis control, reducing usage of anthelmintics and thus delaying the spread of anthelmintic resistance.

*Fasciola hepatica*, the common liver fluke, causes disease and production losses in grazing animals, particularly sheep and cattle. The highly pathogenic immature flukes migrate through the liver, causing damage to the parenchyma, and adult flukes in the bile ducts feed on blood and cause biliary endothelial hyperplasia. Clinical signs of disease can include weight loss, anaemia and sudden death in heavily parasitized animals. Sub-clinical infections cause reduced growth rates in beef cattle and lower milk yields in dairy cows. It is estimated that the global cost of *Fasciola* infection is over $3billion per year^1^. In addition, it has been shown that the disease can adversely affect the results of routine tests for bovine tuberculosis^2^.

The current control of *F. hepatica* relies primarily on the use on anthelmintic drugs designed to kill the parasite. However, resistance to triclabendazole, the drug with greatest efficacy against the immature stages, is evident in fluke populations worldwide and widespread in many sheep-rearing areas of Europe^3^,^4^. A vaccine would greatly enhance control. Several antigens of *F. hepatica*, potential components of a vaccine, have been characterised and their efficacy in inducing protection investigated. These include cathepsin L1 and L2, fatty acid binding protein, glutathione S-transferase, peroxiredoxin and leucine aminopeptidase^5^. Antigens have been tested either alone or in combination, with different adjuvants and delivery systems and in different host species including goats, cattle and sheep. Under experimental conditions, protection has been variable, ranging from 29% to 72% in terms of reduction in worm burden. In one study in which calves were naturally exposed to a low level of infection after vaccination with recombinant cathepsin L1 (rFhCL1), a reduction in worm burden of 48% was observed^6^. In many trials, although vaccination resulted in a relatively modest reduction in worm burden, there was a significantly lower egg output and an anti-embryonic effect^5^. For nematode and tick vaccines, reduced fecundity has a major impact on subsequent infection challenge^7^. However the impact of the anti-fecundity effect in *F. hepatica* is made more complicated since the life cycle relies on an intermediate host (the dwarf pond snail *Galba truncatula*) in which clonal amplification occurs (see Table 1 for a brief description of the stages that occur outside the definitive (mammalian) host). Moreover it is not clear if protection of around 50% in terms of worm burden is sufficient to warrant commercialisation of a vaccine and most prototype vaccines have not been tested in the field.

It is unlikely that, in the foreseeable future, a vaccine will be developed that offers complete protection against *F*. *hepatica*. The aim of this project was, therefore, to develop a model of *F. hepatica* infection that could be used to assess, under simulated field conditions, the effectiveness of vaccines providing incomplete protection against *F*. *hepatica*. Specifically, we assess which potential vaccine effects (reduction in fluke fecundity, increase in fluke maturation time and increase in immature fluke death rate) offer the greatest overall benefit in terms of reducing fluke burden (in the current season) and egg production (potentially affecting pasture contamination the following season). We began by building a mathematical model that describes the acquisition and development of fluke in the definitive host while taking into account heterogeneity in susceptibility between hosts together with seasonal changes in temperature, which affect the development of stages on the pasture. We use the model to assess the efficacy of vaccination and duration of protection required to reduce disease and transmission within a flock or herd in a single season.

## Results

### Model validation

Before using our model to study vaccination, we first verify that it adequately represents the behaviour of unvaccinated herds by comparing the qualitative behaviour of the model against two sets of data.

In reality, when animals are turned out in spring, the pasture is already contaminated with a relatively small number of metacercariae that have survived the winter or been released by infected snails that have successfully overwintered. Typically, this leads to a low level of infection in animals. At the same time, the increase in temperature triggers development and hatching of eggs that have been lying dormant. This hatch leads, via infection of snails, to a large number of metacercariae being released on the pasture in late summer, which in turn generates the large number of infected animals generally seen in autumn.

**Figure 1:**
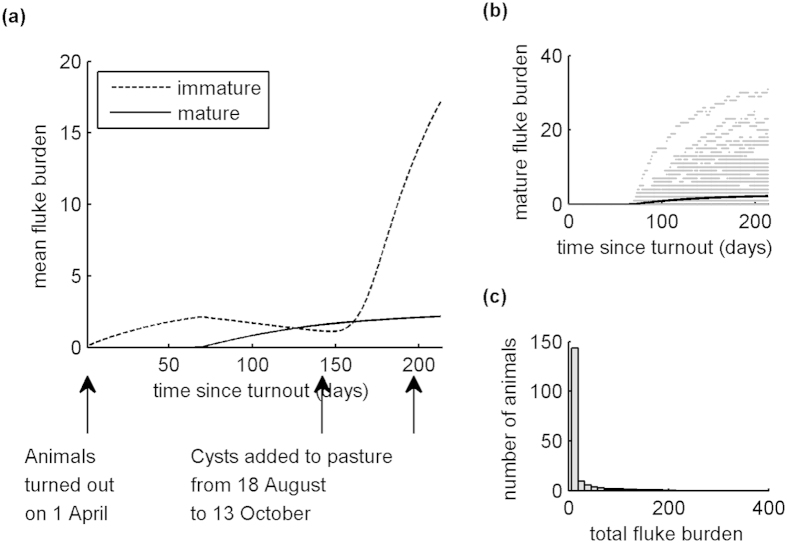
Typical output produced by the stochastic, individual-based model when naïve, unvaccinated animals are turned out on to contaminated pasture on 1^st^ April and followed until 31^st^ October. Initially, the pasture contained 50,000 metacercariae. A further 500,000 were added between 18^th^ August and 13^th^ October. (**a**) shows the change in mean fluke burdens over time, (**b**) shows the change in individual mature fluke burdens (grey) with the change in the mean mature fluke burden (black as in (**a**)), (**c**) shows the mean distribution of total fluke burden on 31^st^ October.

**Figure 2:**
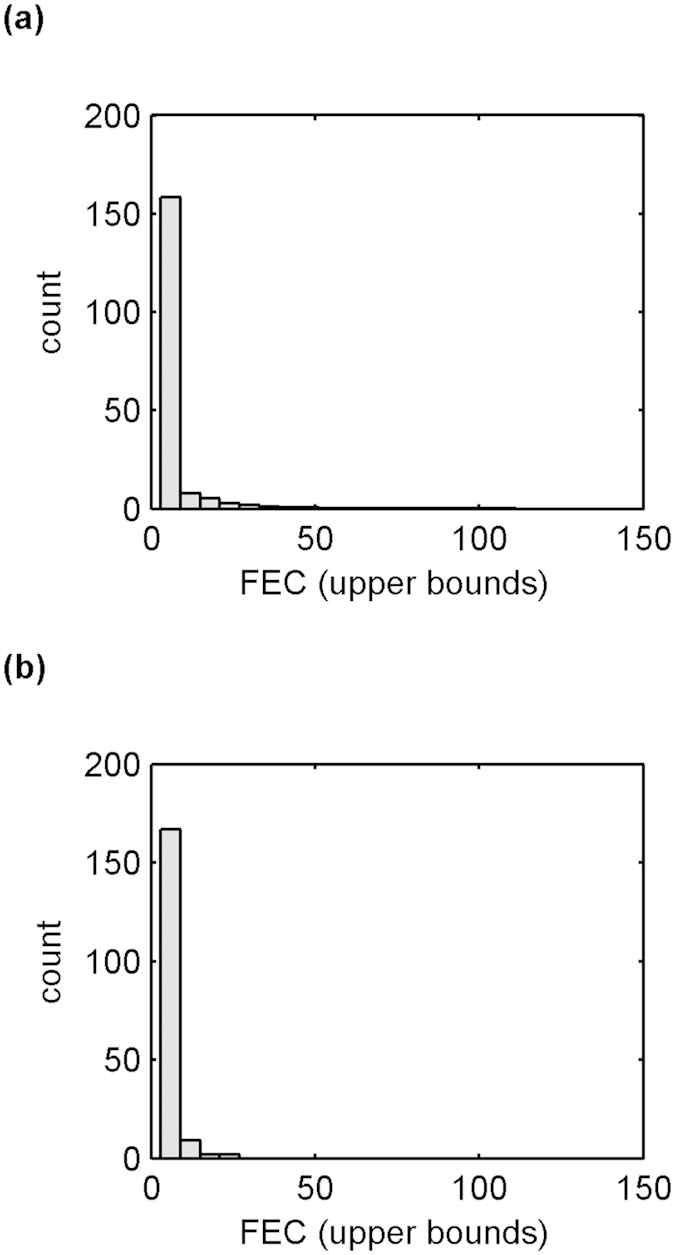
Comparison of model output and faecal egg count data. Distributions of faecal egg counts (**a**) produced by the model for 31^st^ October and (**b**) collected from a single herd of 180 animals in November 2013. Daily egg output from the model was converted into “number of eggs in 2g of faeces” (to match the real data) by multiplying by 2/37100 (because an adult dairy cow produces on average 37.1 kg of faeces per day^30^,^31^). Mean distribution was calculated by averaging across 100 simulations.

Figure 1 shows typical output from our model when naïve, unvaccinated calves are turned out on to contaminated pasture on the 1^st^ April and followed until 31^st^ October. The plot of mean fluke burden against time (Fig. 1a) clearly shows a small peak in spring, after the introduction of the initial 50,000 metacercariae to the pasture, followed by a large peak in autumn after the introduction of a further 500,000 metacercariae over a 56 day period. We can also see from the ‘cloud’ plot (Fig. 1b) and the distribution of total fluke burden (Fig. 1c) that the model generates some individuals with high fluke burdens (maximum typically around 300 flukes), while the majority have burdens close to the mean (19.33). This value is consistent with the value (16.43) quoted by Golden *et al*.^6^ for a naturally-acquired infection. Figure 2 shows the distributions of faecal egg counts (a) produced by the model for 31^st^ October and (b) collected from a single herd of 180 animals in November 2013. The two distributions are comparable and provide further support for the model’s ability to adequately replicate infection dynamics within a herd.

### Effect of vaccine action

**Figure 3:**
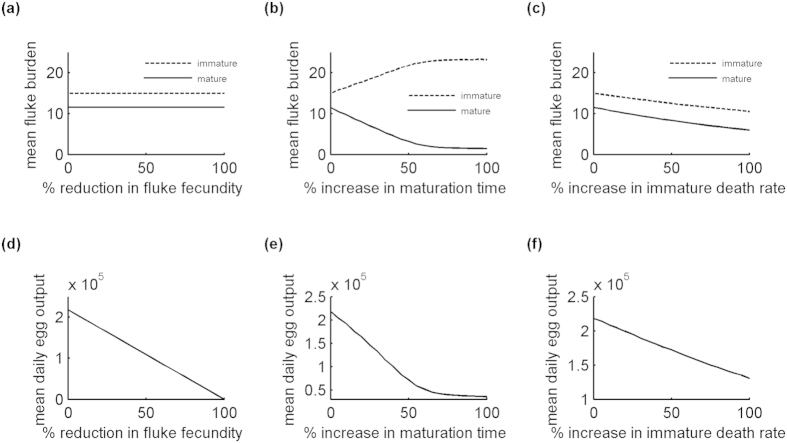
Effect of vaccine action on mean fluke burden and mean daily egg output (i.e. mean number of eggs produced per animal per day) at the end of December for animals turned out on 1^st^ April. (**a,d**) percentage reduction in fluke fecundity (100 × *VE0*_*ff*_). (**b,e**) percentage increase in average fluke maturation time (100 × *VE0*_*fm*_). (**c,f**) percentage increase in immature fluke death rate (100 × *VE0*_*id*_). In each case, the percentage of the population (herd) protected (*cv*) was set to 100% and the duration of protection (*T*) was set to 365 days, ensuring maximum vaccine efficacy until 31^st^ December.

Here we consider a vaccine that provides incomplete protection against *F. hepatica*. It does not prevent infection (i.e. vaccinated hosts are fully susceptible), but can reduce burden and egg output. The vaccine acts on transmission in three ways. It can reduce fluke fecundity (*VE0*_*ff*_), increase average fluke maturation time (*VE0*_*fm*_) and increase immature fluke death rate (*VE0*_*id*_). We examined the impact of each action by running the model 100 times for each of 21 different levels of efficacy. To emphasise any effect, the proportion of the population protected was set to 100% and the vaccine was prevented from waning. Figure 3 shows the effect of each action on the mean fluke burden and mean daily egg output (i.e. mean number of eggs produced per animal per day) recorded at the end of December for animals turned out on 1^st^ April.

Figure 3a,d confirm that reducing fluke fecundity has no effect on the final fluke burden (i.e. the mean burden at the end of December) for the current season, but does reduce final egg output. Increasing the average fluke maturation time reduces the final mature fluke burden, but as a consequence the final immature fluke burden is higher (Fig. 3b). It also reduces final egg output (Fig. 3e). However, the effect starts to level off for increases over 50%. This is because the follow-up time becomes short relative to the increased maturation time. Increasing the immature death rate reduces the final immature fluke burden (Fig. 3c), which leads to a reduction in the final mature fluke burden and final egg output too (Fig. 3f).

### Effect of proportion protected & duration

**Figure 4:**
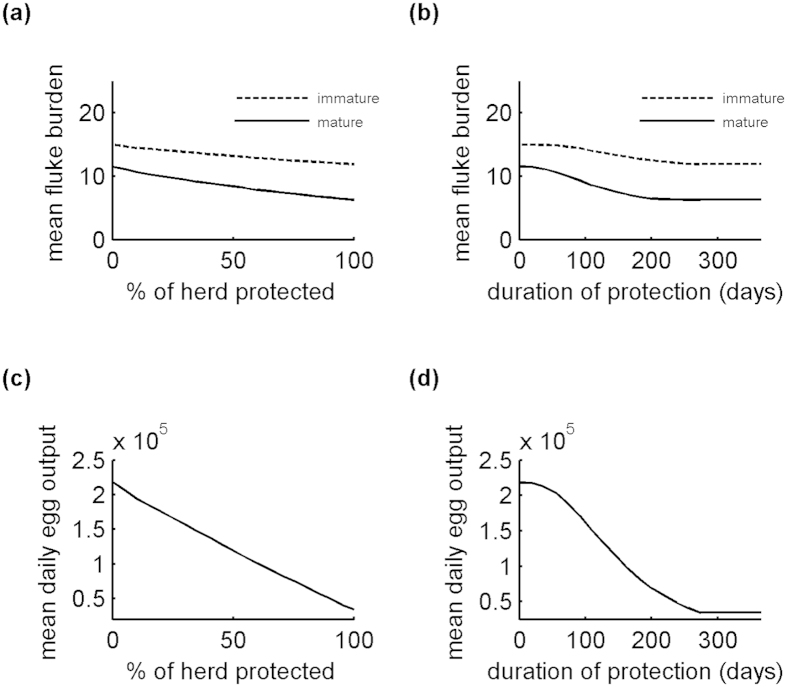
Effect of percentage protected and duration of protection on mean fluke burden and mean daily egg output (i.e. mean number of eggs produced per animal per day) at end of December for animals turned out on 1^st^ April. (**a,c**) percentage of the population (herd) protected (100 × *cv*). (**b,d**) minimum duration of protection (*T*). A minimum duration of 274 days gives complete protection until 31 December. In each case, the vaccine efficacies *VE0*_*ff*_, *VE0*_*fm*_ and *VE0*_*id*_ were set to values approximately equal to the mid-points of their feasible ranges (i.e. 0.75, 0.05 and 0.75, respectively). So, there was a 75% reduction in fluke fecundity, a 5% increase in average fluke maturation time and a 75% increase in immature fluke death rate.

The effects of varying the proportion of the population protected (*cv*) and minimum duration of protection (*T*) are shown in Fig. 4. The vaccine efficacies *VE0*_*ff*_, *VE0*_*fm*_ and *VE0*_*id*_ were set to values approximately equal to the mid-points of their feasible ranges (i.e. 0.75, 0.05 and 0.75, respectively). Note that, as the animals were turned out on 1^st^ April, a minimum duration of 274 days was sufficient to give complete protection until 31 December.

Figure 4 shows that increasing the proportion of the population protected, and similarly the minimum duration of protection, reduces the final fluke burden and, to a greater extent, the final egg output. For burden, the benefit of increasing the minimum duration of protection starts to level off at around 240 days (i.e. after the second peak in immature fluke burden), suggesting that some waning is acceptable. However, for egg output, maximum benefit is achieved with durations of 274 days or more (i.e. with complete protection up to or beyond the end of the study).

**Figure 5:**
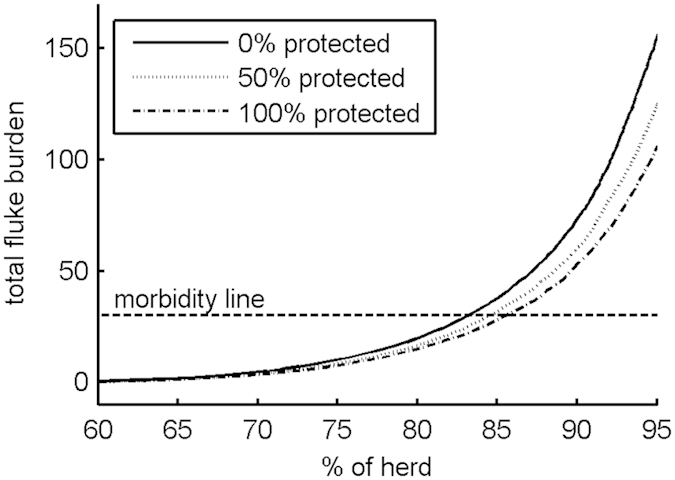
Average cumulative distribution of final total fluke burden for different levels of protection. In each case, the vaccine efficacies (*VE0*_*ff*_, *VE0*_*fm*_ and *VE0*_*id*_) were set to values approximately equal to the mid-points of their feasible ranges, so there was a 75% reduction in fluke fecundity, a 5% increase in average fluke maturation time and a 75% increase in immature fluke death rate. The duration of protection (*T*) was set to 365 days, ensuring maximum efficacy until 31 December. The morbidity line indicates the lowest fluke burden at which production losses have been observed^8^.

With our individual-based model, we are not restricted to considering the effect of the vaccine on *mean* fluke burden or *mean* egg output. We can examine its effect on the distribution of individual animal values. This is illustrated in Fig. 5, which shows the average cumulative distribution of final total fluke burden for different levels of protection (100 × *cv*). The morbidity line indicates the lowest fluke burden at which production losses have been observed^8^. Figure 5 shows how the percentage of the population (herd) with burdens below this economic threshold increases as the level of protection increases.

This figure also reveals that the majority of individuals (>80%) have low fluke burdens in the absence of vaccination (*cv* = 0). This explains why increasing efficacy and duration of protection has less effect when the proportion of the population protected is low (results not shown).

### Sensitivity analysis

The model is described in detail in the Methods section. For convenience, the model and its parameters are also summarised in Table 2. The sensitivity of key model outputs to each of these parameters is shown in Table 3. Sensitivity was measured by calculating partial rank correlation coefficients (PRCC). Parameters with an absolute PRCC value greater than 0.2864 are considered to be important. If the values had been calculated using data rather than simulation results, then the cut off would indicate statistical significance at the 1% level.

The sensitivity analysis reveals that the final immature and mature fluke burdens are greatly influenced by the average maturation time (*τ*_0_), the immature fluke death rate (*μ*_20_), the vaccine-induced increase in the immature fluke death rate (*VE0*_*id*_), the times at which metacercariae start and cease to be added to the pasture (*t*_*e*2_ and *t*_*e*3_), the rate of infection (*β*_1_), the vaccine-induced increase in the average maturation time (*VE0*_*fm*_) and the proportion of the population protected by the vaccine (*cv*). Of these parameters, only the latter was found to be important for final egg output. The final immature fluke burden was also found to be sensitive to two parameters of the metacercaria loss function, namely the maximum possible survival time (*peaksurv*) and *L*_2_ which controls the ‘flatness’ of the metacercaria loss peak. In addition to the parameters listed above, the final mature fluke burden was also greatly affected by the mature fluke death rate (*μ*_3_). In terms of final egg output, only three parameters were found to be important: the density-dependent factor (*z*) that reduces fluke fecundity in individuals with high fluke burden, the vaccine-induced reduction in fluke fecundity (*VE0*_*ff*_) and, as mentioned above, the proportion protected (*cv*). The latter was the only parameter found to affect all three measures of infection. Duration of protection (*T*) was not found to be important, because the minimum value considered within the sensitivity analysis (i.e. 300) was large enough to ensure maximum vaccine efficacy until the end of the study (i.e. 31 December).

### Optimum vaccination strategy

It is unlikely that liver fluke will ever be eradicated. However, with a suitable vaccine it should be possible to reduce transmission and disease. Current prototype fluke vaccines are able to reduce fluke burden and also egg output, thereby reducing contamination of the pasture and presumably transmission to the next generation. So far, we have looked at the effects of the individual actions of our hypothetical vaccine. Here we look for optimum combinations that reduce burden (and hence disease) and egg output (and hence transmission) to acceptable levels.

**Figure 6:**
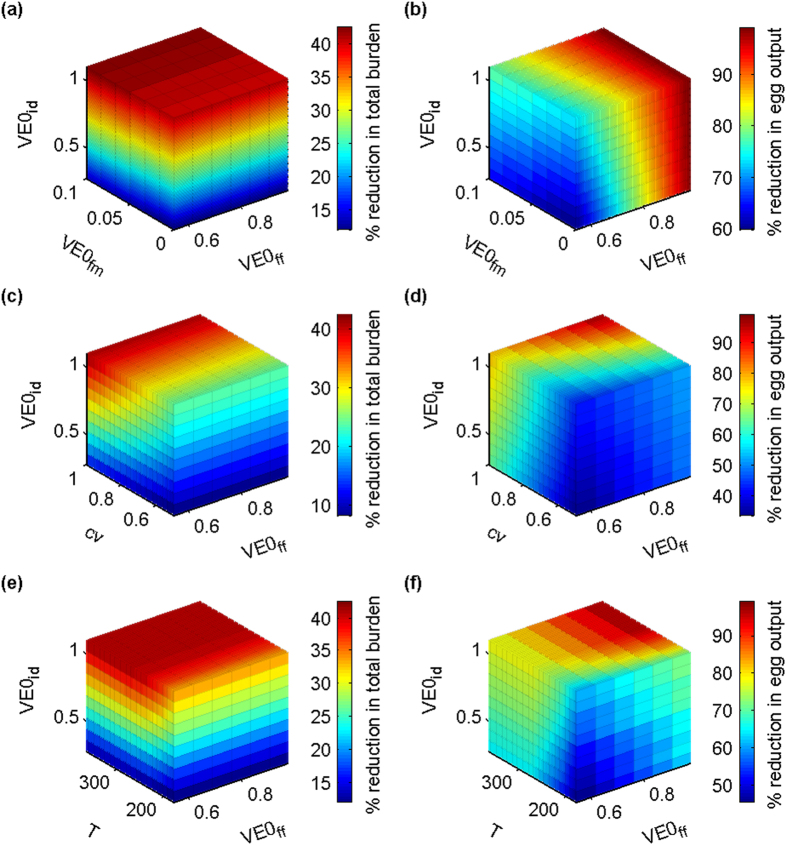
Plots showing the percentage reduction in total fluke burden and daily egg output per host for different combinations of vaccine parameter values. In all of the plots, *VE0*_*ff*_ = 0.55–0.98 and *VE0*_*id*_ = 0.2707–1.1003. The three remaining parameters take the following values. (**a,b**) *VE0*_*fm*_ = 0–0.1, *cv* = 1, *T* = 365. (**c,d**) *VE0*_*fm*_ = 0.1, *cv* = 0.5–1, *T* = 365. (**e,f**) *VE0*_*fm*_ = 0.1, *cv* = 1, *T* = 180–365.

In Fig. 6a,b, the three vaccine efficacy parameters vary across their respective feasible ranges (i.e. *VE0*_*ff*_ = 0.55–0.98, *VE0*_*fm*_ = 0–0.1 and *VE0*_*id*_ = 0.2707–1.1003) while the proportion protected (*cv*) equals 1 (i.e. all animals are protected) and the duration of protection (*T*) is 365 days (i.e. maximum efficacy for duration of study). The colour represents the percentage reduction in total fluke burden (or egg output as appropriate) produced by each combination of values. Figure 6a shows that, in terms of reducing total fluke burden this season, the increase in immature fluke death rate (*VE0*_*id*_) is the most important parameter. The reduction in fluke fecundity (*VE0*_*ff*_) has no effect (as in Fig. 3a) and the increase in fluke maturation time (*VE0*_*fm*_) has very little effect over its feasible range. However, in terms of reducing egg output (Fig. 6b), and hence contamination of the pasture, the most important parameter is *VE0*_*ff*_.

Having identified *VE0*_*ff*_ and *VE0*_*id*_ as important parameters, we then substituted *cv* (proportion protected) [Fig. 6c,d] and *T* (duration of protection) [Fig. 6e,f] in turn for *VE0*_*fm*_. In each case, *VE0*_*fm*_ was set to its maximum feasible value (i.e. 0.1). If we specify that an acceptable response to vaccination would be both a greater than 35% reduction in total fluke burden and a greater than 85% reduction in egg output, then we can see from Fig. 6c–f that we would require a vaccine with the following characteristics: *VE0*_*ff*_ > 0.85, *VE0*_*id*_ > 0.85, *cv* > 0.9 and *T* > 275.

## Discussion

Given the cost of liver fluke infection in terms of production losses, and the growing problem of resistance to flukicides, particularly triclabendazole, it is clear that new approaches to the control of liver fluke are urgently needed. Vaccines may offer a solution. A variety of vaccine candidate antigens, including cathepsin L1 and L2, fatty acid binding protein, glutathione S-transferase, peroxiredoxin and leucine aminopeptidase^5^, have been evaluated in several vaccine trials in sheep, cattle and goats^9^. Efficacy of these vaccine antigens has been determined experimentally in small numbers of housed animals, each of which was first vaccinated, then inoculated with a known number of metacercariae and followed for a fixed number of weeks before being slaughtered to allow enumeration of the fluke burden. One trial has been conducted in which vaccinated animals were allowed to graze contaminated pasture, rather than being challenged experimentally^6^. Although this study provided useful information, the level of pasture contamination was low and the follow up time proved to be relatively short. In consequence, no mature flukes or eggs were counted. Overall, however, studies using both experimental challenge and natural exposure demonstrate that novel vaccines are only likely to offer partial protection with reductions in burdens of between 29% and 72% recorded^10^,^11^,^12^. Hence vaccines are unlikely to completely protect animals from infection by liver fluke and their principle effects are to increase the time needed for flukes to mature, increase the mortality rate of immature flukes and reduce fluke fecundity.

In this paper we have used a modelling approach to examine the impact of these vaccine effects on the fluke burden of infected cattle and the rate of output of fluke eggs to identify the optimum characteristics that a future vaccine should offer, given that induction of sterile immunity is unlikely. Our model is an extension of a model by Smith^13^ to which we added heterogeneity in host susceptibility, stochasticity, vaccination (which can reduce fluke fecundity, increase fluke maturation time and/or increase immature fluke death rate) and seasonality through the seasonal exposure of animals to metacercariae and seasonal changes in temperature affecting the survival of metacercariae on the pasture. The model, therefore, has the ability to take estimates from infection studies and simulate a possible outcome under field conditions. Using our model we have shown that, over its feasible range, the increase in fluke maturation time (*VE0*_*fm*_) has a negligible effect on burden and egg output. However, both the reduction in fluke fecundity (*VE0*_*ff*_) and increase in immature fluke death rate (*VE0*_*id*_) can have a large effect: the former in terms of reducing egg output; the latter in terms of reducing burden within a season.

The most significant finding was that increasing the proportion of the population protected (*cv*) and the duration of protection (*T*) can have a large negative effect on both burden and egg output. Given that the distribution of fluke burden is heavily skewed, with the majority of animals having just a few flukes, it is particularly important that the proportion of the population protected by a vaccine is high, if the vaccine is to be effective. Similarly, Sabatelli *et al*.^14^ showed that vaccines have the greatest effect when targeting individuals with the heaviest worm burden. Identifying individuals within a population that are likely to carry the largest worm burden is not realistic, hence delivering a vaccine that may have imperfect efficacy but can protect >90% of a herd is more likely to be commercially successful than one with greater efficacy but a low level of protection.

According to the literature^6^,^10^,^12^,^15^, a reduction in fluke fecundity of between 55% and 98% has been achieved in infection experiments. In addition the viability of surviving eggs is compromised^16^. Our model has not evaluated the impact of a reduction in egg output on challenge in the subsequent season. To achieve this, the current model would need to be extended to incorporate the free-living and intramolluscan stages of the parasite and the impact of temperature and rainfall on these stages. Models described for other parasites such as hookworm^14^ have incorporated the free living component of the life cycle, but *F. hepatica* is complicated by being a hermaphrodite and also undergoing clonal expansion within the intermediate host; infection of a snail with a single miracidium can lead to production of several thousand cercariae (Allen and Hodgkinson, personal communication). Development of a more complex model will aid in understanding the true impact of a reduction in egg output on levels of infection the following year, although this is complicated by the variation in annual weather patterns, which have a significant impact on metacercarial load on pasture^17^. Historically, however, the commercial take up of vaccines that reduce challenge in the long term rather than prevent disease in a specific animal, has been poor^18^,^19^.

Our analysis suggests that current vaccine candidates have the potential to reduce the mean total fluke burden by as much as 43% (Fig. 6a,c,e) and mean daily egg output by as much as 99% (Fig. 6b,d,f) under field conditions, but that duration of protection is important. In a temperate climate, the peak challenge with metacercariae occurs following the summer months when the environmental conditions are favourable for the development of the free-living and intramolluscan stages of the parasite and the expansion of the snail population. However, infection can also occur early in the season from overwintered metacercariae. This rarely causes disease or overt production losses, but the adult fluke produce eggs, which contaminate the pasture, contribute to summer infection in the snails and ultimately infectious load in the autumn. The model demonstrates that protective immunity must be maintained for the whole season (e.g. at least 274 days for animals followed until 31 December; less for animals grazing from April to October).

In conclusion, novel vaccines, while unlikely to offer complete protection, could contribute substantially towards the control of fasciolosis. In addition, their use could delay the spread of anthelmintic resistance by reducing the use of these drugs.

## Methods

### Infection cycle

The life cycle of liver fluke involves stages that develop within an intermediate host (snail), free living stages and stages within the definitive host (cattle and sheep). The first half of the cycle involves ingestion of metacercariae by the mammalian host, which excyst in the gut to produce immature flukes, which migrate to and then through the liver before maturing in the bile ducts. The mature flukes begin to shed eggs onto pasture approximately ten to twelve weeks after infection of the host^20^. The second half of the cycle involves development of eggs into miracidia, infection of nearby intermediate hosts, further development within the intermediate host and finally shedding of cercariae from the intermediate hosts which encyst as metacercariae on vegetation. The time from infection of the snail with miracidia to shedding of cercariae is approximately six to eight weeks but is temperature dependent^20^. For the purposes of assessing the effect of a prototype vaccine, we focussed on the first half of the cycle (i.e. the processes directly affecting the mammalian host). We also assumed for the purposes of this model that the vaccine will be used to protect first season grazing calves from infection.

### Smith’s model

We used as the basis for our model the deterministic model (equations (1, 2, 3, 4)) published by Smith^13^. The variables of Smith’s model are the total numbers of metacercariae (for which he uses the symbol *C* from cysts) and eggs (*E*) on the pasture and the total numbers of immature (*I*) and mature flukes (*M*) in the population. The model includes the mortality of metacercariae at the rate *μ*_1_ per metacercaria per day, as well as the consumption of metacercariae at the rate *β*_1_ per metacercaria per host per day, where *H* is the number of definitive hosts. Consumption leads to immature flukes within the host (i.e. infection). Immature flukes mature after *τ* days, if they do not die in the meantime, which they do at rate *μ*_2_ per immature fluke per day. Mature flukes die at the rate *μ*_3_ per mature fluke per day. While alive, they produce eggs at the rate *λ*_0_*z*^*m*^ per fluke per day, where *m* is the burden in a particular host and *z* controls the degree of density-dependence. Assuming that the distribution of burdens within the host population can be approximated by a Negative Binomial distribution with aggregation parameter *k*_2_ leads to the expression on the right-hand side of equation (4), which gives the number of eggs produced per day by all flukes in the population. Finally, the step-function *θ* (a purely mathematical construct) is 0 when *t* < *τ* and 1 otherwise.


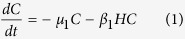



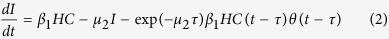



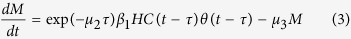



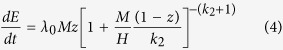


The model follows infection in naive hosts turned out onto contaminated pasture. It is useful because it includes important processes. However, it does not include seasonality or vaccination, nor does it explicitly include heterogeneity in susceptibility between hosts.

### Our model

**Figure 7:**
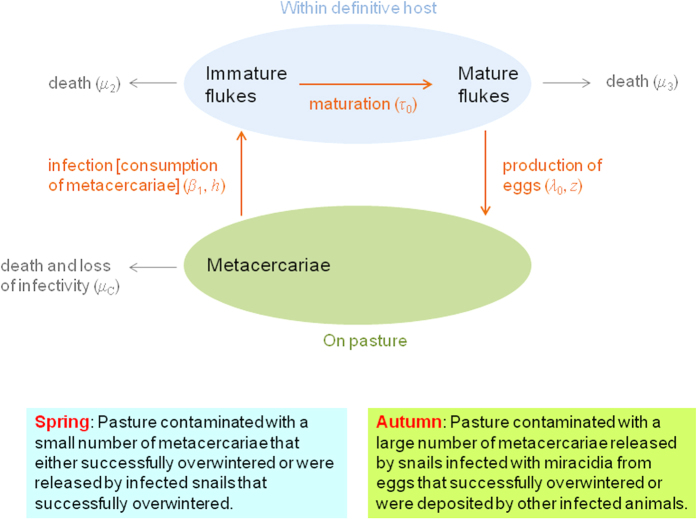
Flow diagram showing the infection model. Infectious metacercariae on the pasture that are not lost are consumed leading to immature flukes within the definitive host (i.e. cattle and sheep). Immature flukes that survive to maturity produce eggs until they die. These eggs are deposited on the pasture in faeces, where they can go on to infect the intermediate host (i.e. the dwarf pond snail *Galba truncatula*). Further development within the intermediate host eventually leads to shedding of cercariae, which encyst as metacercariae on the pasture. The development from eggs on the pasture, through infection of the intermediate host, to the addition of metacercariae to the pasture is not included in our model and hence not shown in the diagram.

To incorporate the additional features listed above, we constructed a stochastic, individual-based, discrete-time model. Essentially, we took Smith’s model and added heterogeneity (i.e. individual animals, each with their own ‘susceptibility’ and hence fluke burden), stochasticity (essential when modelling small populations), vaccination (which can reduce fluke fecundity, increase fluke maturation time and/or increase immature fluke death rate) and seasonality (i.e. seasonal addition of metacercariae to the pasture and temperature-dependent mortality of metacercariae on the pasture). Further details are given below and in Table 2. The model is illustrated in Fig. 7. Note that, in our model, *I* and *M* are the numbers of immature and mature flukes in a particular animal, rather than in the population as a whole as in Smith’s model. Also, we do not model the absolute number of eggs on the pasture. We only record the daily egg output per host.

#### Heterogeneity

Our model describes infection within a herd of *H* animals. By modelling each animal separately, we are able to look at individual fluke burdens as well as the mean value. At the start, each animal is assigned a host susceptibility factor *h* drawn from a Gamma distribution with mean 1 and variance 1/*k*_2_. This approach was used by Sabatelli *et al*.^14^ to model human hookworm infection; and these authors remark that, in combination with birth-death processes, this results in a Negative Binomial distribution of burdens, a distribution commonly associated with helminth infections^21^,^22^. A factor of one corresponds to average susceptibility. By contrast, an individual with a factor of ten would be expected to acquire a burden that was approximately 10 times greater than the herd average. The susceptibility factor represents differences between hosts that can be due to differences in innate susceptibility or exposure.

#### Stochasticity

Herd size *H* can take any value. For the purposes of this study, it was set to 180 animals. Incorporating stochasticity is essential when modelling small populations. We introduced it by sampling from either a Binomial or Poisson distribution, as described below. At each daily time step, the following calculations were made for each animal.

The number of immature flukes acquired at time *t* was drawn from a Poisson distribution with mean equal to the average number that would be acquired in a day by an animal with host susceptibility *h* (i.e. mean = *β*_1_*Chdt*, where *β*_1_ is the rate of infection, *C* is the number of metacercariae at time *t* − 1 and *dt* is the timestep (in this case, 1 day)). So, at each time step a new cohort of immature flukes was created for each host. Each of these flukes was assigned a maturation time that was drawn from a truncated-Normal distribution with a mean equal to the average maturation time *τ*_0_ and a standard deviation of 1.25.

For each host, the number of immature flukes that die at time *t* was drawn from a Binomial distribution with parameters equal to the number present at time *t* − 1 minus the number that mature at time *t* and the probability of dying during a time step (i.e. 1 − exp(−*μ*_2_*dt*), where *μ*_2_ is the mortality rate for immature flukes). The numbers of mature flukes and metacercariae that died at time *t* were calculated in a similar way (see Table 2).

**Figure 8:**
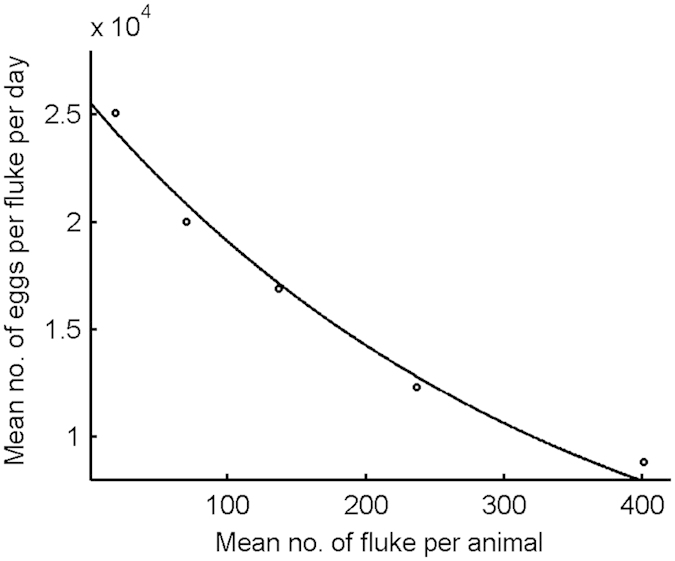
Relationship between the mean number of eggs produced per fluke per day and the mean number of flukes per animal. Note that the relationship is not defined for zero flukes.

The number of eggs produced at time *t* by a host with *M* adult flukes was drawn from a Poisson distribution with mean equal to *Mλ*_0_*z*^*M*^, where *λ*_0_ is the number of eggs produced per day by 1 mature fluke in the absence of density-dependent constraint and *z* controls the degree of constraint. An inverse relationship between egg production and burden has been noted for liver fluke in sheep^23^, but not for cattle. However, inverse relationships have been documented for other helminths including *Haemonchus contortus* in sheep^24^ and *Ostertagia ostertagi* in cattle^25^. Therefore, we chose to include a density-dependent constraint in cattle based on data for sheep. The parameters *λ*_0_ and *z* were estimated by fitting the line *y* = *λ*_0_exp(−*δx*) to data from Happich & Boray^23^, where *y* equals the mean number of eggs per fluke per day and *x* equals the mean number of flukes per animal. Note that *z* = exp(−*δ*). The relationship between the mean number of eggs produced per fluke per day and burden (*M*) is shown in Fig. 8. The relationship is only defined for burdens greater than or equal to one.

#### Vaccination

According to the literature^6^,^10^,^11^,^12^,^15^, the prototype vaccines have three putative modes of action. Hence, we introduced three vaccine efficacy parameters, *VE0*_*ff*_, *VE0*_*fm*_ and *VE0*_*id*_, to represent the reduction in fluke fecundity (*λ*_0_), increase in average fluke maturation time (*τ*_0_) and increase in immature death rate (*μ*_2_) in vaccinated animals. The proportion of the population that is protected (*cv*) was allowed to vary. Also, the duration of protection varied between animals. After the minimum duration of protection had expired, the vaccine efficacy parameters would become zero with probability *dt*/*T*. This process produces a distribution of durations that is equivalent to exponential waning at the population level.

#### Seasonality

**Figure 9:**
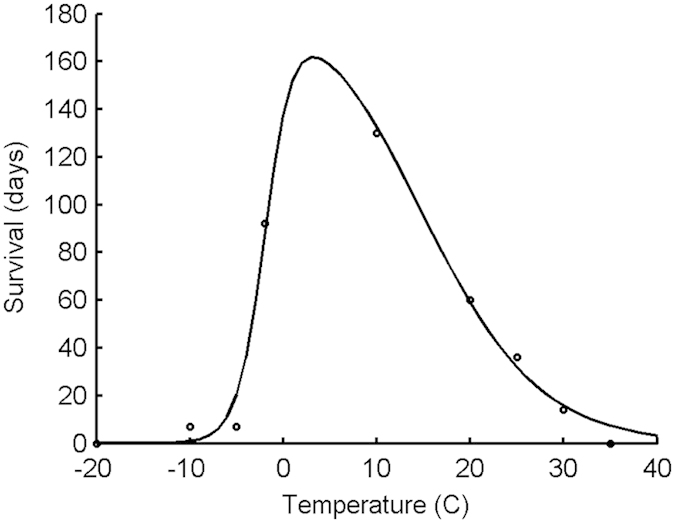
Relationship between temperature and survival of infectious metacercariae.

All parts of the fluke life cycle occurring outside the definitive host are affected by temperature and, in most cases, rainfall too. To incorporate these seasonal effects into our model, we replaced the mortality rate for metacercariae on pasture with a temperature-dependent function. The function was derived by fitting a non-symmetric curve to survival data published by Boray & Enigk^26^ (Fig. 9). As we were only interested in the survival of infectious metacercariae, each data point is the greatest time (in days) for which at least 50% of the metacercariae remained both alive and infectious (indicated by their ability to infect at least 4 out of 5 mice). The mortality rate at time *t* is then determined by the temperature at time *t*, which was generated by a periodic function derived from temperature data. For validation purposes, we used a periodic function based on monthly mean temperatures from 2007–2011 for the postcode area associated with the farm from which the egg count data was obtained (described in the Validation section). For all other simulations, we used a function based on daily mean temperatures for the whole of England and Wales from 2000–2013, downloaded from the website of the UK’s Meteorological Office (Exeter, UK). For details, see Table 2. Further seasonality was introduced by adding metacercariae to the pasture in a way that was consistent with observed contamination. Based on one study in Japan, Smith^27^ concluded that most cercariae emerged within a 2–4 week period each year. Unpublished data from the Netherlands from 1998 and 2004–2009 (kindly provided by Cor Gaasenbeek) reveals that metacercariae were found from April–November with, in six out of seven years, the majority of metacercariae being recorded between August and November. In our model, 50,000 metacercariae were added to the pasture at the time the animals were turned out (*t*_*e*1_). A further 500,000 metacercariae were added over 56 days starting on day *t*_*e*2_ = 230 (i.e. 18^th^ August) and ceasing on day *t*_*e*3_ = 286 (i.e. 13^th^ October). The number added each day gradually increased, peaking in the middle of September before decreasing again, and followed a Normal distribution. The numbers of metacercariae added at the two periods were chosen to represent the low contamination associated with spring and the high level of contamination witnessed in autumn.

### Validation

The model was validated by comparing the output in the absence of vaccination with the mean burden quoted by Golden *et al*.^6^ for a naturally-acquired infection and the distribution of faecal egg counts obtained as follows.

Faecal samples were taken per rectum from 180 adult dairy cattle on a farm in Mid-Wales, in November 2013. The cattle had grazed on pasture known to be at risk of contamination with *Fasciola hepatica* metacercariae. Eggs were isolated from faeces using a Flukefinder® (Richard Dixon, ID, US). This is a commercially available kit comprising a unit made of two 5 cm-wide sieves of approximately 125 nm and 30 nm mesh (exact size proprietary). 2 g of faeces from each individual sample was mixed with water and poured into the Flukefinder® unit and washed well with water. Larger material was retained by the large diameter sieve and discarded. Material retained in the smaller diameter sieve was back washed into a 5 cm beaker. This was allowed to settle for two minutes before the supernatant was poured off. The sedimentation process was repeated until the supernatant was clear. The remaining material was poured into a 5 cm petri dish, methylene blue added and examined under a dissecting microscope. Fluke eggs were counted. Flukefinder® has a sensitivity and specificity comparable to other sedimentation methods^28^. The collection of faecal samples from cattle was carried out in accordance with approved guidelines and regulations (ethical approval no. VREC181 from the University of Liverpool’s Ethical Review Procedure).

### Analysis

The behaviour of the model was analysed by running numerical simulations for different scenarios and parameter sets (100 simulations in each case). A sensitivity analysis was also conducted. This involved calculating partial rank correlation coefficients (PRCC) for each parameter of the model^29^. For the sensitivity analysis, parameters were sampled from their respective feasible ranges. If no range was specified, then values within the range of + /−10% of the point estimate were used. The proportion protected (*cv*) and the duration of protection (*T*) were allowed to vary between 0.8–1 and 300–365 respectively.

## Additional Information

**How to cite this article**: Turner, J. *et al*. A model to assess the efficacy of vaccines for control of liver fluke infection. *Sci. Rep*. **6**, 23345; doi: 10.1038/srep23345 (2016).

## Figures and Tables

**Table 1:** Terms used to describe the various stages of *Fasciola hepatica* that occur outside the definitive (mammalian) host.

Term	Description
Eggs	Shed by infected definitive (mammalian) hosts
Miracidia	Short-lived, motile stages that emerge from eggs and go on to infect intermediate (snail) hosts
Cercariae	Motile stages that are shed by infected intermediate hosts. Clonal amplification within the intermediate host results in many cercariae per miracidium.
Metacercariae	Encysted cercariae that are consumed by mammalian hosts while grazing.

**Table 2:** Details of the stochastic, individual-based model for liver fluke.

Quantity	Function	Parameters
Host susceptibility factor *h*	Gamma (*a*,*b*) with *a* and *b* such that mean = 1, var = 1/*k*_2_^14^	*k*_2_ = aggregation parameter = 0.1
*I*_*A*_ = number of immature flukes acquired at time *t*	Poisson (*β*_1_*Chdt*)	*β*_1_ = rate of infection = 0.000001^27^ *C* = number of metacercariae at time *t* − 1*h* = host susceptibility for that particular individual*dt* = timestep = 1 day
*I*_*D*_ = number of immature flukes that die at time *t*	Binomial (*I*_*t *− 1_ – *I*_*M*_, *p*_*D*_) with *I*_*t *− 1_ = number present at time *t *− 1, *I*_*M*_ = number that mature at time *t* and *p*_*D*_ = probability of dying during a time step	Maturation times (assigned at acquisition) drawn from truncated-Normal distribution with mean = *τ*_*0*_ and standard deviation = 1.25. *τ*_0_ = average fluke maturation time = 70 days (49–84 days)^10^ *μ*_2_ = mortality rate for immature flukes (i.e. exponential decay rate after assuming all metacercariae become immature flukes and all losses occur at immature stage) = 0.0126 (0.0081–0.0151)^10^,^32^*p*_*D*_ = probability of dying = 1 − exp (−*μ*_2_*dt*).
*M*_*D*_ = number of mature flukes that died at time *t*	Binomial (*M*_*t *− 1_, *p*_*D*_) with *M*_*t *− 1_ = number present at time *t *− 1 and *p*_*D*_ = probability of dying during a time step	μ_3_ = mortality rate for mature flukes (i.e. 1/lifespan) = 0.0028 (0.00069−0.0037) (i.e. average = 12 months, range = 9 months−4 years)^33^*p*_D_ = probability of dying = 1 . exp (−μ_3_dt).
*C*_*D*_ = number of metacercariae that died at time *t*	Binomial (*C*_*t *− 1_, *p*_*D*_) with *C*_*t *− 1_ = number present at time *t *− 1 and *p*_*D*_ = probability of dying during a time step	*μ*_C_ = mortality rate for metacercariae (see below)*p*_*D*_ = probability of dying = 1 − exp (−*μ*_C_*dt*).
*E*_*M*_ = number of eggs produced by a host with *M* adult flukes	Poisson (*Mλ*_*0*_*z*^*M*^)	*λ*_0_ = number of eggs produced by 1 mature fluke in the absence of density-dependent constraint (i.e. when *z* = 1) = 25600*z* = 0.997073 (controls degree of constraint)^23^
*λ*_*V*_ = fluke fecundity in protected animals	*λ*_0_ (1 − *VE0*_*ff*_)	*VE0*_*ff*_ = reduction in fluke fecundity = (0.55–0.98)^6^,^10^,^12^,^15^
*τ*_*V*_ = average fluke maturation time in protected animals	*τ*_*0*_ (1 + *VE0*_*fm*_)	*VE0*_*fm*_ = increase in average fluke maturation time = (0–0.1)^12^
*μ*_*2V*_ = immature fluke death rate in protected animals	*μ*_*2*_ (1 + *VE0*_*id*_)	*VE0*_*id*_ = increase in immature fluke death rate (estimated using vaccine-induced reduction in burden and immature fluke mortality rate *μ*_2_) = (0.2707–1.1003)^10^,^11^,^12^
*μ*_*C*_ (*temp*) = mortality rate for metacercariae at temperature *temp*	1/{(*peaksurv*/4) (1 − tanh((*L*_1_ − *temp*)/*T*_1_)) (1 − tanh((*temp* − *L*_2_)/*T*_2_))}	*peaksurv* = maximum possible survival time = 193.3 d*L*_1_ = −1.871 (no biological interpretation)*L*_2_ = 14.84 (flatness increases with |*L*_2_–*L*_1_|) *T*_1_ = 3 (rise is steeper than descent if *T*_1_/*T*_2_ < 1)*T*_2_ = 12.5^26^
*temp* = temperature at time *t*	*temp* = *meanTemp* + *T*_*a*_[sin(*θ*(*t *+* ϕ*))]	*meanTemp* = annual mean temperature in °C = 8.824 (for validation), 10.48 (for other simulations)*T*_*a*_ = amplitude of temperature fluctuation = 5.573 (for validation), 6.36 (for other simulations) *θ* = scales cycle length = 0.0173 (to 364d for validation), 0.0172 (to 365d for other simulations)*ϕ* = shifts peak in temperature = −130.1169 (to 9^th^ August for validation), −116.2 (to 26^th^ July for other simulations)

**Table 3:** The sensitivity of the stochastic, individual-based model to changes in model parameters was assessed by calculating partial rank correlation coefficients (PRCC values) for three outcome variables, namely mean immature fluke burden (mean I), mean mature fluke burden (mean M) and mean egg output (mean Eout).

Parameter description	Parameter	PRCC values		
		mean I	mean M	mean Eout
average maturation time	*τ*_0_	**0.9310**	**−0.9632**	−0.1733
immature fluke death rate	*μ*_20_	**−0.8783**	**−0.9016**	−0.0437
increase in immature death rate	*VE0*_*id*_	**−0.8220**	**−0.8420**	0.0188
time metacercariae cease to be added to pasture	*t*_*e*3_	**0.8043**	**−0.7987**	−0.0974
time metacercariae start to be added to pasture	*t*_*e*2_	**0.7842**	**−0.8209**	0.0173
rate of infection	*β*_1_	**0.6805**	**0.5742**	−0.0379
maximum possible survival time	*peaksurv*	**0.5236**	0.1272	0.0769
metacercaria loss curve parameter 2	*L*_2_	**0.5203**	0.1661	0.0980
increase in fluke maturation time	*VE0*_*fm*_	**0.4372**	**−0.5556**	0.0379
proportion protected	*cv*	**−0.3278**	**−0.2994**	**−0.3171**
mature fluke death rate	*μ*_3_	0.0354	**−0.4814**	−0.1994
density-dependent factor	*z*	0.0170	−0.0741	**0.9606**
reduction in fluke fecundity	*VE0*_*ff*_	0.1430	0.2381	**−0.4569**
fluke fecundity	*λ*_0_	0.1148	0.0717	0.0067
metacercaria loss curve parameter 3	*T*_1_	−0.1110	0.0404	−0.0813
number of animals	*H*	−0.1077	−0.0773	0.2301
degree of aggregation	*k*_2_	−0.0782	0.2321	0.0005
metacercaria loss curve parameter 1	*L*_1_	0.0436	−0.1737	−0.1052
time vaccination applied and turned out	*t*_*v*1_	−0.0424	0.0826	0.1149
minimum duration of protection	*T*	0.0176	−0.0781	−0.1837
metacercaria loss curve parameter 4	*T*_2_	−0.0175	−0.1401	−0.2845

Parameters associated with an absolute PRCC value greater than 0.2864 are considered to be important. If the values had been calculated using data rather than simulation results, then the cut off would indicate statistical significance at the 1% level.

## References

[b1] Spithill T. W., Smooker P. M. & Copeman D. B. *Fasciola gigantica*: Epidemiology, control, immunology and molecular biology in Fasciolosis (ed. Dalton J. P.) 465–525 (CABI Publishing, 1999).

[b2] Claridge J. . *Fasciola hepatica* is associated with the failure to detect bovine tuberculosis in dairy cattle. Nat. Comms. 3, 853, doi: 10.1038/ncomms1840 (2012). PMC3989536 22617293

[b3] Daniel R. . A composite faecal egg count reduction test to detect resistance to triclabendazole in *Fasciola hepatica*. Vet. Rec. 171, 153–157, doi: 10.1136/vr.100588 (2012). 22791519

[b4] Hodgkinson J., Cwiklinski K., Beesley N. J., Paterson S. & Williams D. J. L. Identification of putative markers of triclabendazole resistance by a genome-wide analysis of genetically recombinant *Fasciola hepatica*. Parasitology 140, 1523–1533, doi: 10.1017/S0031182013000528 (2013). 23721579

[b5] McManus D. P. & Dalton J. P. Vaccines against the zoonotic trematodes *Schistosoma japonicum*, *Fasciola hepatica* and *Fasciola gigantica*. Parasitology 133, S43–S61, doi: 10.1017/S0031182006001806 (2006). 17274848

[b6] Golden O. . Protection of cattle against a natural infection of *Fasciola hepatica* by vaccination with recombinant cathepsin L1 (rFhCL1). Vaccine 28, 5551–5557, doi: 10.1016/j.vaccine.2010.06.039 (2010). 20600503

[b7] Smith W. D. & Zarlenga D. S. Developments and hurdles in generating vaccines for controlling helminth parasites of grazing ruminants. Vet. Parasitol. 139, 347–359, doi: 10.1016/j.vetpar.2006.04.024 (2006). 16750599

[b8] Vercruysse J. & Claerebout E. Treatment vs non-treatment of helminth infections in cattle: defining the threshold. Vet. Parasitol. 98, 195–214 (2001). 11516586 10.1016/s0304-4017(01)00431-9

[b9] Molina-Hernández V. . Fasciola hepatica vaccine: we may not be there yet but we’re on the right road. Vet. Parasitol. 208, 101–111, doi: 10.1016/j.vetpar.2015.01.004 (2015). 25657086 PMC4366043

[b10] Dalton J. P., McGonigle S., Rolph T. P. & Andrews S. J. Induction of protective immunity in cattle against infection with *Fasciola hepatica* by vaccination with cathepsin L proteinases and with hemoglobin. Infect. Immun. 64, 5066–5074 (1996). 8945548 10.1128/iai.64.12.5066-5074.1996 PMC174490

[b11] Mulcahy G. . Correlation of specific antibody titre and avidity with protection in cattle immunized against *Fasciola hepatica*. Vaccine 16, 932–939 (1998). 9682340 10.1016/s0264-410x(97)00289-2

[b12] Mulcahy G. . Immune responses of cattle to experimental anti-*Fasciola hepatica* vaccines. Res. Vet. Sci. 67, 27–33 (1999). 10425237 10.1053/rvsc.1998.0270

[b13] Smith G. Density-dependent mechanisms in the regulation of *Fasciola hepatica* populations in sheep. Parasitology 88, 449–461 (1984). 6739131 10.1017/s003118200005472x

[b14] Sabatelli L., Ghani A. C., Rodrigues L. C., Hotez P. J. & Brooker S. Modelling heterogeneity and the impact of chemotherapy and vaccination against human hookworm. J. R. Soc. Interface 5, 1329–1341, doi: 10.1098/rsif.2007.1255 (2008). 18331978 PMC2607437

[b15] Guasconi L. . Immunization with crude antigens plus aluminium hydroxide protects cattle from *Fasciola hepatica* infection. J. Helminthol. 86, 64–69, doi: 10.1017/S0022149X11000022 (2012). 21366935

[b16] Chryssafidis A. L., Fu Y., De Waal T. & Mulcahy G. Standardisation of egg-viability assays for *Fasciola hepatica* and *Calicophoron daubneyi*: A tool for evaluating new technologies of parasite control. Vet. Parasitol. 210, 25–31, doi: 10.1016/j.vetpar.2015.03.005 (2015). 25863897

[b17] Ollerenshaw C. B. & Rowlands W. T. A method of forecasting the incidence of fascioliasis in Anglesey. Vet. Rec. 71, 591–598 (1959).

[b18] de la Fuente J. . A ten-year review of commercial vaccine performance for control of tick infestations on cattle. Anim. Health Res. Rev. 8, 23–28, doi: 10.1017/S1466252307001193 (2007). 17692140

[b19] Miller R. . Exploring the use of an anti-tick vaccine as a tool for the integrated eradication of the cattle fever tick, Rhipicephalus (Boophilus) annulatus. Vaccine 30, 5682–5687, doi: 10.1016/j.vaccine.2012.05.061 (2012). 22687762

[b20] Scottish Agricultural College. Treatment and Control of liver fluke in Sheep and Cattle. Technical Note 557 (ISBN 1 85482 798 7) (2003).

[b21] Churcher T. S., Ferguson N. M. & Basáñez M. G. Density dependence and overdispersion in the transmission of helminth parasites. Parasitology 131, 121–132, doi: 10.1017/S0031182005007341 (2005). 16038403

[b22] Shaw D. J., Grenfell B. T. & Dobson A. P. Patterns of macroparasite aggregation in wildlife host populations. Parasitology 117, 597–610 (1998). 9881385 10.1017/s0031182098003448

[b23] Happich F. A. & Boray J. C. Quantitative diagnosis of chronic fasciolosis. 2. The estimation of daily total egg production of *Fasciola hepatica* and the number of adult flukes in sheep by faecal egg counts. Aust. Vet. J. 45, 329–331 (1969). 5817299 10.1111/j.1751-0813.1969.tb05012.x

[b24] Roberts J. L. & Swan R. A. Quantitative studies of ovine haemonchosis. I. Relationship between faecal egg counts and total worm counts. Vet. Parasitol. 8, 165–171 (1981). 7201195

[b25] Smith G., Grenfell B. T. & Anderson R. M. The regulation of *Ostertagia ostertagi* populations in calves: density-dependent control of fecundity. Parasitology 95, 373–388 (1987). 3696771 10.1017/s0031182000057814

[b26] Boray J. C. & Enigk K. Laboratory studies on the survival and infectivity of *Fasciola hepatica*- and *F. gigantica*-metacercariae. *Z*. Tropenmed. Parasitol. 15, 324–331 (1964). 14316630

[b27] Smith G. An analysis of variations in the age structure of *Fasciola hepatica* populations in sheep. Parasitology 84, 49–61 (1982). 7063254 10.1017/s0031182000051659

[b28] Faria R. N., Cury M. C. & Lima W. S. Evaluation of two available methods to detect eggs of *Fasciola hepatica* in cattle faeces. Arq. Bras. Med. Vet. Zootec. 60, 1023–1025 (2008).

[b29] Blower S. M. & Dowlatabadi H. Sensitivity and uncertainty analysis of complex models of disease transmission: an HIV model, as an example. Int. Stat. Rev. 62, 229–243 (1994).

[b30] ADAS. Managing Livestock Manures, Booklet 1, 2nd Edition (eds Chambers B. .) (2001).

[b31] Universities Federation for Animal Welfare. Management and Welfare of Farm Animals, The UFAW Farm Handbook 4th Edition (eds Ewbank R., Kim-Madslien F. & Hart C. B.) (1999).

[b32] Brockwell Y. M., Spithill T. W., Anderson G. R., Grillo V. & Sangster N. C. Comparative kinetics of serological and coproantigen ELISA and faecal egg count in cattle experimentally infected with *Fasciola hepatica* and following treatment with triclabendazole. Vet. Parasitol. 196, 417–426, doi: 10.1016/j.vetpar.2013.04.012 (2013). 23643623

[b33] Boray J. C. Experimental fascioliasis in Australia. Adv. Parasit. 7, 95–210 (1969). 10.1016/s0065-308x(08)60435-2 4935272

